# A Systematic Review of Heart Rate Variability as a Measure of Stress in Medical Professionals

**DOI:** 10.7759/cureus.34345

**Published:** 2023-01-29

**Authors:** Jeremy E Peabody, Rebecca Ryznar, Markus T Ziesmann, Lawrence Gillman

**Affiliations:** 1 General Surgery, University of Manitoba, Winnipeg, CAN; 2 Biomedical Sciences, Rocky Vista University College, Colorado, USA; 3 General Surgery/Critical Care, University of Manitoba, Winnipeg, CAN

**Keywords:** acute medical emergency management, biometric sensor, systematic literature review, stress, heart rate variability (hrv)

## Abstract

Understanding the physiological effects of responding to crises is a critical component in understanding how to manage and prepare medical professionals to be crisis responders. Heart rate variability (HRV) is the variation in rate between a succession of R-R intervals. This variation is not only affected by physiological processes such as respiration or metabolic rate but is also directly controlled by the autonomic nervous system. As such, heart rate variability has been proposed as a noninvasive tool to measure the physiological stress response.

The aim of this systematic review is to consolidate heart rate variability literature in the context of medical emergencies to determine if heart rate variability changes predictably from baseline when responding to medical crises. This may demonstrate utility as an objective, noninvasive measure of stress response.

A systematic literature review of six databases yielded 413 articles, 17 of which met our inclusion criteria of being written in English, measuring HRV in healthcare providers, and measuring HRV in real or simulated medical resuscitations or procedures. Articles were then analyzed using the Grades of Recommendation, Assessment, Development, and Evaluation (GRADE) scoring system.

Out of the 17 articles reviewed, 11 demonstrated statistically significant results showing heart rate variability responding in a predictable manner to stress. Three articles utilized a medical simulation as the stressor, six used medical procedures, and eight used medical emergencies encountered during clinical work. Overall, a predictable trend in heart rate variability metrics of standard deviation from the mean value of normal-to-normal (N-N) intervals (SDNN), root mean square of the successive differences (RMSSD), mean number of times per time interval in which the change in successive normal sinus (N-N) intervals exceeds 50 ms (PNN50), low frequency % (LF%), and low-frequency-to-high-frequency ratio (LF/HF) was observed when responding to stress.

This systematic literature review showed that heart rate variability among healthcare providers responding to stressful scenarios follows a predictable pattern of change and expands our understanding of the physiology of stress in healthcare providers. This review supports the use of HRV to monitor stress during high-fidelity simulation to ensure that appropriate physiological arousal is achieved during the training of medical personnel.

## Introduction and background

The field of medicine is filled with challenging situations and critical decisions that must be made rapidly, often by medical personnel fatigued or emotionally stressed. When responding to an emergency, many factors contribute to well-studied physiological stress responses including increased serum cortisol, blood pressure elevation, and elevated heart rate [1-6]. The State-Trait Anxiety Inventory (STAI) score represents a subjective interpretation of stress experienced but fails to capture physiological changes related to stress. Recently, heart rate variability (HRV) has become commonly used as a reliable, noninvasive method of measuring physiological responses to stress.

The mechanism by which the heart responds to stress is well understood. The heart contains intrinsic pacemaker cells that regulate its rate and rhythm. However, these cells can be influenced by the autonomic nervous system to accommodate the body’s hemodynamic requirements when a stressor is introduced. In the absence of physiological or extrinsic stressors, the parasympathetic nervous system (PNS) primarily influences the pacemaker cells of the heart [7]. Beat-to-beat variability in one’s heart rate is the result of competing PNS and sympathetic nervous system (SNS) responses that are balanced to meet the body’s changing hemodynamic requirements. As the SNS drive causes the heart rate to increase, the beat-to-beat interval is reduced so there is less time between heartbeats and less variability; thus, HRV decreases. When the PNS has a predominant influence on the sinoatrial node, heart rates are lower and variability increases. This is called cycle length dependence [8,9]. Because of this variability, groups such as the Task Force of the European Society of Cardiology (ESC) and the North American Society of Pacing and Electrophysiology (NASPE) in 1996 have identified heart rate variability as a physiological marker of stress-induced elevation in SNS and reduction in PNS [8].

When measuring HRV, there are multiple variables that can be analyzed. These variables can be divided into three main domains: time domain, frequency domain, and geometric domain.

Time domain

The time domain measures HRV over time intervals between normal cardiac cycles. This is commonly measured by calculating the time between successive q-waves on an electrocardiogram (ECG). Throughout the literature, this value is represented as the mean normal-to-normal (N-N) interval. For statistical analysis, the standard deviation from the mean value of N-N intervals is used (SDNN). When looking at the numerical values of SDNN and its relationship to HRV, the larger the SDNN, the greater the HRV. As previously stated, the greater the HRV, the more influence the PNS has on the heart’s automaticity.

Another common time value that is used in the analysis is the root mean square of the successive differences (RMSSD). This value assesses the root mean square of the proportion of N-N intervals that are greater than 50 ms to the total number of N-N intervals. The RMSSD represents the PNS, and a larger RMSSD corresponds to increased HRV (Table 1).

**Table 1:** Heart rate variability time domain metric explanations adapted from the Task Force of the European Society of Cardiology and the North American Society of Pacing and Electrophysiology *N-N interval = R-R intervals on ECG ^A^Standard deviation between N-N intervals ^B^Root mean square of the standard deviation between N-N intervals ^C^Number of adjacent N-N intervals varying by >50 ms ^D^Mean number of times in which the change in successive N-N intervals exceeds 50 ms N-N interval: normal-to-normal (N-N) interval, ECG: electrocardiogram

Variable	Unit	Description
SDNN^A^	ms	The standard deviation of all N-N intervals*
RMSSD^B^	ms	The square root of the mean of the sum of the squares of the difference between adjacent N-N intervals
NN50 count^C^		The number of pairs of adjacent N-N intervals differing by more than 50 ms in the entire recording; three variants are possible: counting all such N-N intervals or only pairs in which the first or second interval is longer
PNN50^D^	%	NN50 count divided by the total number of all N-N intervals

Frequency domain

HRV may be analyzed from the frequency domain, where data quantifies the contribution from each of the PNS and the SNS. The term used to describe the analysis of HRV in the frequency domain is the power spectral density (PSD). At baseline, a physiological phenomenon known as respiratory sinus arrhythmia (RSA) occurs, resulting in R-R interval variation created by the respiratory cycle. The vagal parasympathetic effect slows the heart rate during expiration, creating more variation (as per the cycle length dependence phenomenon described above). During inspiration, the vagal drive is diminished, ending the RSA. The high-frequency (HF) band (0.15-0.40 Hz) captures the RSA resulting from PNS vagal stimulation from both central and reflex interactions. The low-frequency (LF) band (0.04-0.15 Hz) measurements capture information during inspiration, during which the cardioinhibitory vagal stimulation is inhibited. Studies have combined the two bands to give a measurement of the low-frequency-to-high-frequency ratio (LF/HF) to represent the proportion of PNS/vagal inhibition relative to PNS/vagal stimulation (Table 2) [4-6,8,10-15].

**Table 2:** Heart rate variability frequency metric explanations adapted from the Task Force of the European Society of Cardiology and the North American Society of Pacing and Electrophysiology ^a^Low frequency ^b^Low frequency in normalized units ^c^High frequency ^d^High frequency in normalized units ^e^Low-frequency-to-high-frequency ratio Hz: Hertz, VLF: very low frequency

Variable	Unit	Description
LF^a^	Hz	Power in the low-frequency range (0.04-0.15 Hz)
LF (norm)^b^	Normalized units	Low-frequency power in normalized units: low frequency/(total power-VLF) × 100
HF^c^	Hz	Power in the low-frequency range (0.15-0.40 Hz)
HF (norm)^d^	Normalized units	High-frequency power in normalized units: high frequency/(total power-VLF) × 100
LF/HF^e^		Ratio of low frequency (ms^2^)/high frequency (ms^2^)

Geometric domain

The geometric domain is represented by the heart rate variability triangular index (HTI). This measure calculates the integral of the density of the R-R interval histogram divided by its height. A five-minute epoch is conventionally used to represent this metric. Combining the HTI and RMSSD values can distinguish normal from dysrhythmic heart rhythms [15].

HRV has been used in a number of both simulation and real-life studies looking at participants during crises in the medical literature. Understanding the psychophysiological stress response among medical learners can have many implications for future medical training. Although it is easy to understand that medical emergencies will create a stress response among medical professionals, to our knowledge, there are no published systematic reviews that assess the changes in HRV in medical professionals responding to medical emergencies. The goal of this paper is to perform a systematic review of literature that summarizes and critically evaluates the body of evidence to answer the following question: among medical professionals responding to medical emergencies, do HRV metrics change in a predictable way?

The abstract of this article was previously presented as a poster in the Critical Care Canada Forum in 2020.

## Review

Methods

Search Strategy

Excerpta Medica database (EMBASE) (Ovid Technologies, Inc., Elsevier Science Publishers), The National Library of Medicine (PubMed and Medical Literature Analysis and Retrieval System Online (Medline)), Cumulative Index of Nursing and Allied Health Literature (CINAHL) database, National Institute of Health database, and Scopus were searched for articles written in English published between 1990 and 2020 pertaining to HRV changes in healthcare personnel when responding to real or simulated medical emergencies and real or simulated procedures. When searching each of the databases, the following combinations of search terms were used: (1) EMBASE: (“Title = (“Heart Rate Variability” OR “HRV”) AND Title =(“medical Emergencies” OR “Code Blue” or “Resuscitation”)), (2) PubMed: (“Heart rate variability (title) AND Health Personnel (title/MESH) AND Medical Emergencies (title/MESH) NOT “Morbidity” NOT “survival),” (3) Medline: (“heart rate variability” (keyword) AND “medical” (all fields) and “emergency” (all fields)), (4) CINAHL: (“Title = heart rate VARIABILITY” AND Title = “Health Personnel” AND Title = “Medical Emergencies” NOT Title = “Mortality” NOT Title = “Pediatrics”), (5) National Institute of Health: (“HEART RATE VARIABILITY (all fields) AND Medical Emergencies (all fields)), and (6) Scopus: (heart AND rate AND variability AND medical AND emergencies AND health AND personnel). The reference lists for any review articles found in the initial search, as well as citations from eligible studies, were mined for additional studies.

Study Selection

Utilizing two reviewers (LG and JP), a two-step review of all articles returned by our search strategies was performed. First, the reviewers independently screened all titles and abstracts of the returned articles to decide if they met the inclusion criteria. Second, the full text of the chosen articles was then assessed to confirm if they met the inclusion criteria. Studies measuring HRV in medical personnel responding to an acute medical emergency or ill patient during a real or simulated event were included in the review. Studies were excluded if they involved pediatric subjects and subjects who were not medical care providers or if HRV was measured during sleep or as a marker of chronic stress. Any discrepancies between the two reviewers were resolved by a third party (MZ).

Quality of Evidence

The two independent reviewers graded the included studies using the Grades of Recommendation, Assessment, Development, and Evaluation (GRADE) approach [16]. The GRADE approach has four levels of quality of evidence, with the highest quality rating given to a randomized study. Observational studies have a low-quality rating but can be upgraded to a moderate-quality rating or downgraded to a very low-quality rating based on similar factors such as the risk of bias, consistency of results, and magnitude of effect.

Data Collection and Synthesis of Results

Data were extracted from the eligible studies and tabulated in electronic form. A narrative synthesis was felt to be the most appropriate approach since there were multiple data fields being examined, and this allowed the reviewers to ensure that the context of the study aligned with the inclusion criteria. The data fields extracted were as follows: study goal, study population, the setting during which monitoring took place, HRV variables reported, type/make of monitor used, and major findings or results. A summary of the extracted data and GRADE evaluations is available in Table 3.

**Table 3:** Summary of the literature review HRV: heart rate variability, HR: heart rate, VLF: very low frequency, LF: low frequency, HF: high frequency, LF/HF: low-frequency-to-high-frequency ratio, Ln LF/HF: natural logarithm of low-frequency-to-high-frequency ratio, RMSSD: root mean square of the successive differences between normal heartbeats, TP: total spectral power (overall autonomic activity), AVNN: average N-N interval, PNN50: mean number of times per time interval in which the change in the successive normal sinus (NN) intervals exceeds 50 ms, GRADE: Grades of Recommendation, Assessment, Development, and Evaluation, BP: blood pressure, PGY: postgraduate year, PTSD: post-traumatic stress disorder, D: diurnal, N: nocturnal, ASs: attending surgeons, JRs: junior residents, SRs: senior residents, HLTA: high-level trauma activation, MLTA: mid-level trauma activation, HEMS: helicopter emergency medical services, CEA: carotid endarterectomy, MWL: mental workload, AUC: area under the curve

First author, year	Method	Sample	Stressor	Measure	Outcome	Device used		GRADE justification
Schneider et al., 2017 [1]	To confirm the validity of HR and HRV in prehospital care by emergency room physicians (cross-sectional design)	12 anesthesiologists and one surgeon in the role of emergency physicians (pre-hospital primary care) (Germany)	Responding to pre-hospital patients requiring the sorties	SDNN, RMSSD, PNN50, LF, HF, LF/HF	Mean difference between pre-alarm phase versus primary care phase: statistically significant difference for SDNN, RMSSD, PNN50, HF, and LF/HF, ꝉ = statistically significant (P < 0.05)	Zephyr’s BioHarness 3.0 (Zephyr Technology, Annapolis, MD, USA)		Nonexperimental design, small sample size, lack of generalizability due to it being a single-center study, consistent and significant results, GRADE: moderate
Henriksen et al., 2018 [2]	To quantify physician stress levels when performing lumbar puncture (LP) and explore operator stress effect on patient outcomes	46 physicians/medical students (emergency medicine and neurology): 22 novices, 12 intermediates, and 12 experts (three outpatient neurology clinics in Denmark)	Lumbar puncture procedure	Ln LF/HF * mean difference between during and pre-lumbar puncture	Statistically significant difference in Ln LF/HF between intermediate and novice pre-procedure, no statistically significant difference between groups while doing the procedure, ꝉ = statistically significant (P < 0.05)	Faros 180° sensor (Mega Electronics Ltd., Kuopio, Finland)		Nonexperimental design, small sample size, imprecise measure of subjective stress, potential for response bias, several professions involved, GRADE: very low
Aasa et al., 2006 [3]	To assess physiological and subjective markers of stress in ambulance personnel during both working and nonworking hours (cross-sectional design)	26 ambulance personnel (Sweden)	Continuous monitoring for 72 hours (24 hours on duty and 48 hours off duty)	Mean value LF spectral power (%)	Stress markers did not show differences between the work shift and leisure time, modest deviation in HRV pattern during work in comparison with work-free days observed in personnel with many health complaints, no statistically significant differences	DL700 Holter recorder (Braemar Inc., Burnsville, MN, USA)		Nonexperimental design, small sample size, GRADE: low
Petrowski et al., 2018 [4]	To compare the stress of emergency physicians on helicopter emergence medical services on regular shifts compared to control days (cohort design)	20 emergency physicians on the HEMS team (German Air Rescue Team)	HEMS day versus clinic day versus control, HEMS responds to medical emergencies in the field	HF RMSSD, LF/HF, SDNN	Δ air versus clinical: statistically significant difference for LF/HF metric only, Δ air versus control: no statistically significant differences, Δ clinic versus control: statistically significant difference in SDNN metric only; ꝉ = statistically significant (P < 0.05)	Zephyr’s BioHarness 3.0 (Zephyr Technology, Annapolis, MD, USA)		Nonexperimental design, small sample size, lack of generalizability due to the specificity of the few helicopter physicians working, GRADE: very low
Ghazali et al., 2018 [5]	To determine if technical and nontechnical performances correlated during immersive simulations and to quantify the physiological markers of stress (cross-sectional study)	48 participants divided into 12 interdisciplinary teams (emergency physicians, ambulance drivers, medical residents, and registered nurses) (French National Health and Medical Research Institute)	High-fidelity simulations (pediatric patient with hypovolemic shock)	Δ PNN50, Δ LF/HF	Mean Δ PNN50 immediately before simulation-immediately after simulation: 9.0 ꝉ P < 0.0001, mean Δ LF/HF immediately before simulation-after simulation: -3.5 ꝉ P < 0.0001, ꝉ = statistically significant (P < 0.05)	Continuous electrocardiographic record (Holter analysis with the software Syncope) (Sorin Group, Clamart, France)		Nonexperimental design, small sample size, GRADE: low
Oldenburg et al., 2014 [6]	To measure psychomental stress/strain among fire department dispatchers during emergency situations (randomized control trial)	47 participants: 27 in the experimental arm (call dispatchers) (Europe) and 20 in the control arm (administrative work)	Responding to 1-2-2 calls (emergency services in Europe)	SDNN, LF/HF, HR	Mean Δ SDNN experimental-control (ms): statistically significant for events and no events, mean Δ LF/HF experimental-control: no statistically significant difference, mean Δ HR experimental-control (bpm): statistically significant differences for events, no events, and breaks, ꝉ = statistically significant	Portable telemetric beat-to-beat recorder Polar RS 800 with 1,000 Hz sampling rate (Polar Electro, Oy-Kempele, Finland)		Experimental designs, participants were aware of which group they were in, comparison to subjective measure with a chance of response bias, GRADE: low
Zenati et al., 2019 [10]	To illustrate an example of how MWL data may be incorporated into root cause analysis of clinical incidents (case report)	Attending anesthesiologist, attending surgeon, lead perfusionist	A near-miss incident during a coronary artery bypass graft surgery	LF/HF	Anesthesiologist, attending surgeon, and perfusionist: LF/HF (AUC) pre-procedure versus during procedure: no statistically significant difference	Polar H10 sensors (Polar Electro Inc., Finland)		Nonexperimental design, very small sample size, GRADE: very low
Adams et al., 1998 [11]	To determine if the HRV and BP of emergency physicians are affected during a night shift (cross-sectional design)	12 emergency medicine attending physicians (Northwestern Hospital USA, 1989-1991)	Stress during a night shift in the emergency department	R-R interval, LF, HF, LF/HF	Δ pre-shift - during shift, no statistically significant differences	Marquette 8000T Laser Holter monitor (Marquette Electronics, Inc., Milwaukee, WI, USA)		Nonexperimental design, small sample size, GRADE: low
Baker et al., 2016 [12]	To objectively and subjectively compare responses in a simulated environment compared to an operating theater environment (cohort study)	8 anesthesia residents	Uncomplicated rapid sequence intubation	LF/HF	Δ mean LF/HF simulation versus theater environment: 4.47 higher in theater ꝉ p = 0.006, Δ mean LF/HF simulation versus baseline: no statistically significant difference, p = 0.86, ꝉ = statistically significant (p < 0.05)	Wireless Polar RS800CX monitor© (Polar Electro 2011, Warwick, UK)		Nonexperimental design, small sample size, GRADE: low
Joseph et al., 2016 [17]	To assess the level of stress subjectively (via State-Trait Anxiety Inventory and NASA Task Load Index) and objectively via HRV in trauma team members (attendings and residents) (cohort study)	22 participants (ASs: n = 8, JRs: n = 7, SRs: n = 7) (Arizona level 1 trauma center)	Activations of the trauma team: HLTA, MLTA, and emergency surgeries	Duration of time spent with HRV below baseline (high-level stress = HRV < 65% baseline), mean duration of stress duration during HLTA and emergency surgeries	Mean stress duration during shift: ASs = 13.52%, SRs = 70.12%, JRs = 71.46%; JR duration HLTA: 71.22, SR HLTA: 61.98, AS: 27.07; JR emergency surgery: 79.69, SR emergency surgery: 82.30, AS emergency surgery: 42.31; statistically significant difference between ASs and both groups of residents in HLTA and emergency surgeries; no statistically significant difference between the duration of stress between JRs and SRs during HLTA or emergency surgery	Zephyr’s BioHarness 3.0 (Zephyr Technology, Annapolis, MD, USA)		Nonexperimental design, small sample size, dose-response effect, lack of generalizability due to it being a single-center study, GRADE: very low
Rodrigues et al., 2018 [18]	To assess the level of stress firefighters face during situations they are called to respond to (cross-sectional study)	17 firefighters (Portugal)	Accidents, pre-hospital assistance (BP control, glycemia control, etc.), fires	AVNN, LF/HF	Mean AVNN (ms) accidents, pre-hospital: no statistically significant difference, mean LF/HF (ms) accidents, pre-hospital, mean LF/HF fires (ms): no statistically significant difference, ꝉ = statistically significant	VitalJacket®		Nonexperimental design, small sample size, lack of generalizability due to it having a single population grouping, overconsumption, GRADE: very low
Gallo et al., 2012 (abstract only) [19]	To determine if stress response change with more experience in performing medical procedures	21 medical students and emergency medicine residents	Endotracheal intubation	SDNN, VLF, TP, LF	Mean Δ of SDNN, TP, or LF (students versus residents): no statistical difference, mean Δ PGY1 versus PGY3 VLF: no statistical difference	Not stated		Nonexperimental design, small sample size, lack of generalizability due to it being a single-center study, methods section is missing information, GRADE: very low
Ghazali et al., 2019 [20]	To follow HRV response to daily simulations and determine if repetitive high-fidelity simulations create PTSD in participants (cohort study)	48 participants in (12) multidisciplinary emergency medicine teams (physician, resident, nurse, and ambulance driver)	Managing infant shock in a high-fidelity simulation center: experimental group, nine simulations over one year; control group, three simulations over one year	Mean Δ LF/HF at each stage of the experiment	LF/HF experimental initial: D = 78.03 ± 7.49 and N = 2.93 ± 1.26, LF/HF experimental intermediate: D = 4.72 ± 2.12 and N = 3.48 ± 1.99, LF/HF experimental final: D = 4.55 ± 2.27 and N = 3.21 ± 1.39; LF/HF control initial: D = 4.89 ± 2.05 and N = 3.15 ± 1.86, LF/HF control intermediate: D = 5.18 ± 3.21 and N = 4.05 ± 2.95, LF/HF control final: D = 5.64 ± 3.42 and N = 3.92 ± 2.86; no statistically significant differences, ꝉ = statistically significant (P < 0.05)	Holter monitor with Syncope (Sorin Group) software		Nonexperimental design, small sample size, GRADE: low
Mefford et al., 2019 [21]	To determine if HRV can serve as a real-time index of autonomic arousal and if it can serve as a measure of the effectiveness of stress-modifying interventions	8 PGY1 medical trainees in emergency medicine during their anesthesia rotation (64 intubations were studied) (USA)	First intubation of the day	Δ RMSSD from baseline	Mean Δ RMSSD from baseline prior to intubation versus during intubation: no statistically significant difference; statistically significant difference between RMSSD two minutes prior to intubation and during intubation versus baseline	Not stated		Nonexperimental design, very small sample size, methods section is missing information, GRADE: very low
Stein, 2020 [22]	To measure the objective stress and subjective anxiety of medical trainees performing a simulation (randomized control trial)	36 students from all four years of Bachelor of Health Sciences in emergency medical care in Johannesburg, South Africa	Experimental group: simulation, control group: no simulation	SDNN, RMSSD, HF	Median Δ SDNN, Δ RMSSD, Δ HF experimental versus control: no statistically significant difference		HR monitor (Actiheart, CamNtech, Cambridge, UK)	Experimental design, lack of generalizability due to it being a single-center study, easy to distinguish control versus experimental group, GRADE: low
Wetzel et al., 2010 [23]	To investigate stress levels and coping strategies of surgeons in simulated surgeries (randomized control trial)	30 participants (residents in general or vascular surgery and attending physicians of vascular surgery), two groups: low experience = 2-6 years of surgical experience, high experience ≥ 6 years of surgical experience	2 simulated CEA procedures: 1: no complications, 2: complications	SDNN, HRV coefficient (C_HRV) was calculated: C_HRV = (SDNN/NN) × 100 LF/HF	Δ mean SDNN, C_HRV, LF/HF from baseline: no statistically significant difference		Wireless HR monitor (S801i, Polar, Kempele, Finland)	Experimental design, small sample size, lack of generalizability due to it being a single-center study, GRADE: moderate

Results

**Figure 1:**
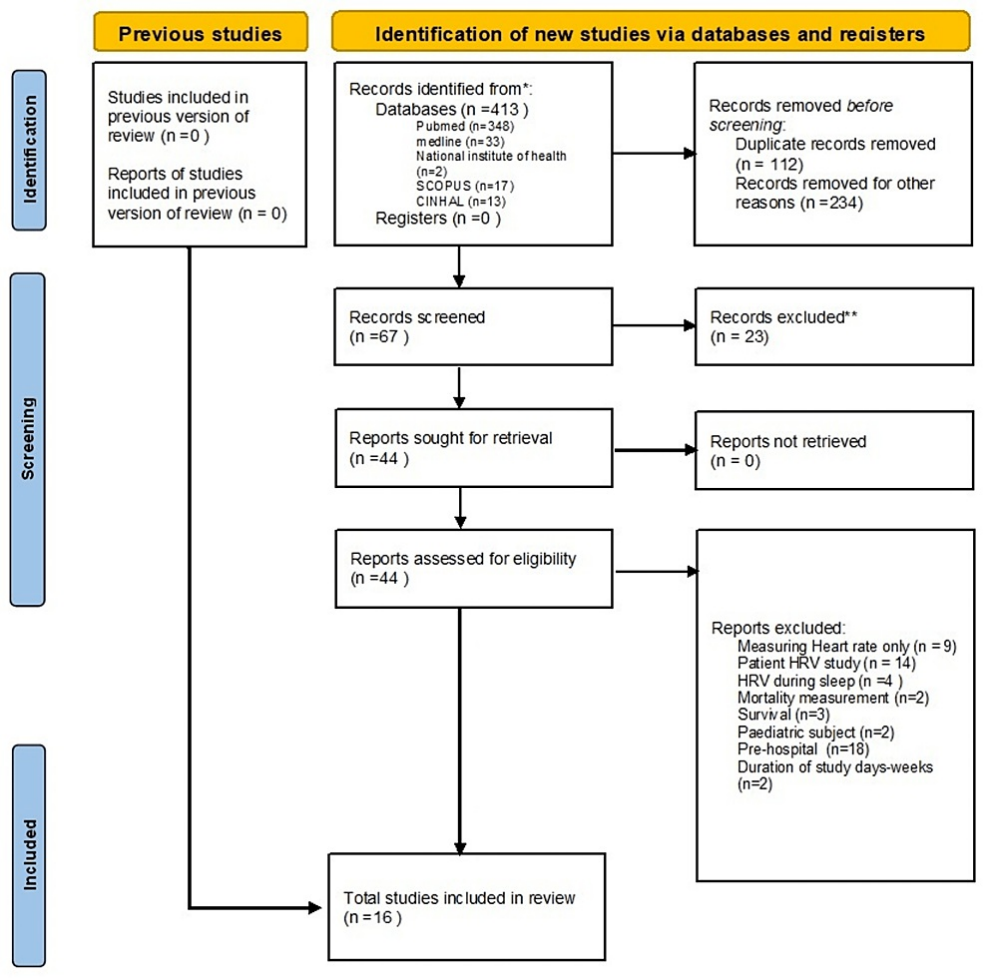
Summary of search results from the search strategy HRV: heart rate variability

A total of 413 articles were identified based on the initial search strategy. Of these, 67 articles were selected after screening titles and abstracts, and duplicates were removed. After a full-text review, 16 articles fit the inclusion criteria (Figure 1).

Three of the articles reviewed studied HRV changes in participants using simulated emergencies. Six articles assessed HRV during procedures such as intubations and lumbar punctures, and eight articles measured HRV during emergencies encountered during a shift. Of these 17 articles that were reviewed, eight reported statistically significant changes in HRV from baseline when participants were responding to medical emergencies [1,2,4-6,13,17,18]. Although they did not all show statistically significant differences, all the studies reviewed showed that HRV metrics (SDNN, RMSSD, PNN50, LF%, and LF/HF) responded to the stress of a medical emergency in a similar way. A summary of the literature reviewed can be seen in Table 3.

HRV has been proven to be a valid and reliable noninvasive technique to measure stress in healthcare providers responding to medical emergencies. The literature shows a common theme that HRV metrics respond similarly to stressful situations in each of the studies reviewed.

Within the time domain, the HRV variables SDNN, PNN50, and RMSDD were commonly reported. When included, SDNN and RMSSD consistently showed a reduction in response to being involved in a medical emergency [1,4-6,21-23]. This indicates less frequent ectopic beats and less variability in the cardiac cycle due to an increased sympathetic nervous system drive [1,15,24]. These findings can be generalized beyond medical emergencies as SDNN is sensitive to distinguishing between different mental workloads/stress levels [25].

Within the frequency domain, the LF/HF proved to be statistically significant most consistently of all the frequency domain variables reported [1,2,4,5,11,22]. All studies that examined the LF/HF ratio in response to medical emergencies showed an increase in the LF/HF ratio when encountering medical emergencies [1-6,10-13,18-20,22,23]. A number of studies included in this review found a link between the time domain and the frequency domain through the SDNN and LF power bands. Given that these variables are indicative of sympathetic drive, this relationship demonstrates the understanding that mental stress contributes significantly to sympathetic nervous system activation [26-28]. Looking at both the time and frequency domains together, it has been shown that HRV metrics do change in a predictable manner in healthcare personnel in response to medical emergencies. These results are as predicted: it has been well documented that medical emergencies are stressful for physicians, nurses, and trainees alike [3,25-30].

Discussion

Prior to using HRV to measure the body’s physiological response to stress, methods such as salivary cortisol tests and urine catecholamines were used. These markers were also measured in some of the studies included in this literature review. In general, serum cortisol showed a common response when compared to HRV metrics in response to stress. Three out of the four studies assessing serum cortisol showed statistically significant changes in serum cortisol levels in response to the stress of a medical emergency [3-6]. Serum cortisol, although reliable, is costly, cumbersome to obtain and analyze, and susceptible to physiological diurnal fluctuations, leading to errors in interpretation [19]. Urine catecholamines and heart rate elevation were also evaluated as physiological markers of stress. These markers are not ideal measurements of physiological response to stress because they are not specific. Urine catecholamines lack the ability to provide specific feedback about the medical emergency since it represents the physiological changes that occur between urine collections [6,17]. Heart rate elevation, as previously discussed, can be influenced by several variables such as physical exertion and previous stress, making it not ideal for measuring stress in medical personnel responding to medical emergencies [1,3,6,17,18]. HRV proves to be a better method for understanding physiological response to stress as it shows the timing of the stressor, and through the frequency domain, the data can distinguish from physical strain versus sympathetic drive elevation in response to a stressful situation such as medical emergencies [14,17].

Changes in heart rate, blood pressure, urine catecholamines, and serum or salivary cortisol are all proven methods of evaluating stress; however, a great deal can be missed if the subjective experience is not evaluated also. Out of the studies that were included in this literature review, several used the State-Trait Anxiety Inventory (STAI). The STAI is understood to be the gold standard for measuring situational anxiety [31,32]. The studies that included the STAI showed a reciprocal correlation with HRV. As the STAI score increased, which indicated a situation of greater stress, the HRV decreased in response to that situation [2,3,25,33]. With this correlation, it can be seen that there is a dose-response relationship between the subjective stress of a situation and the HRV reduction due to greater activation of the sympathetic nervous system. This relationship would be shown best by correlating subjective stress with the LF/HF ratio, seeing as how this ratio indicates the relative sympathetic to parasympathetic activation.

These studies included participants from a variety of medical fields including firefighters, nurses, attending physicians, and medical residents from various subspecialties, each of which showed similar HRV changes when the participants underwent a stressful situation. Interestingly, studies in which participants were more physically active (e.g., firefighters responding to fires or performing extractions at car crashes) did not show a significant difference in HRV metrics when responding to these stressful events compared to more sedentary stressful situations (i.e., intubations and medical simulations) [18]. Prospective observational studies showed that heart rate changes associated with the stress of call shifts and working in emergency settings or on helicopter rescue teams carry over through subsequent days up to 72 hours even when the participants were in less stressful settings such as in clinics or on days off [15,18,34]. This leads to the conclusion that HRV is a more sensitive and specific metric of stress than heart rate alone. This means that the metrics used in these studies (AVNN and LF/HF ratio) are less skewed by physical exertion in the same way that heart rate would be. Although all medical professions included in the literature review showed a predictable response in their HRV metrics, Henriksen et al. (2018) [2] and Joseph et al. (2016) [17] demonstrated that there was a statistically significant difference between the HRV metrics in attending level physicians (with greater experience) compared junior and senior residents when responding to medical emergencies. These results reveal that the autonomic nervous system’s response to stress changes with the level of experience, and therefore, medical personnel with greater experience will demonstrate lower levels of sympathetic nervous system activation. As a result, this may explain why some studies did not show statistically significant changes in HRV metrics in the more senior/experienced medical personnel. This also implies that the stress response may become habituated with multiple exposures to stressful situations. This stress response habituation may represent the rationale behind stress inoculation training [35].

In high-stress occupations such as first responders, trauma surgeons, and military personnel, measuring heart rate variability can help with assessing whether or not habituation has occurred. Medical personnel are vulnerable to the harmful effects of emotional trauma, which are in addition to the risk of burnout [29,30]. Habituation is essential to reduce the deleterious effects of chronic stress. Habituation was first described in the 1960s and describes a form of learning in which the extent of the response to a particular stimulus decreases with repeated exposure to that stimulus. The process of habituation has been shown to be associated with decreases in the magnitude of hypothalamic-pituitary-adrenal (HPA) activity following subsequent exposure to the same stress stimulus [36,37]. Chronic stress is associated with an increased likelihood to develop major depressive disorder, anxiety disorders, and PTSD. Chronic stress without habituation is also associated with poor overall physiological health; for example, it has been shown that stress promotes visceral fat accumulation, subcutaneous fat loss, and cardiovascular dysfunction. On the other hand, habituators are much more protected against cardiovascular complications [38,39]. This process of habituation can be monitored and screened in high-stress occupations by tracking heart rate variability over time and therefore potentially prevent the harmful effects of chronic stress at work.

Relevance

This literature review demonstrates that HRV is a valid and reliable method of assessing physiological responses to stressful situations. The implications of this are quite broad. HRV can be used to evaluate the fidelity of simulations in medical training to ensure that optimal conditions are met to give trainees the most realistic experience possible. Given that experience reduced changes in HRV when responding to medical emergencies, further research can be conducted to determine how many high-fidelity simulations medical trainees need to be a part of before they have reduced stress effects. Once this number is identified, it can serve as a goal for medical education institutions and residency programs to strive for to achieve their goal of graduating competent medical professionals. As technology advances, more discrete forms of heart rate monitors and ECGs have been developed that are capable of data transfer to smartwatches, smartphones, tablets, etc., which may allow HRV to be used in biofeedback training in medical and nonmedical fields alike to combat situational anxiety.

Limitations

This literature review was designed to understand how the HRV of medical personnel changes in response to medical emergencies. HRV itself was used as a physiological marker of stress. However, there are several sub-variables that are included in HRV. In an ideal setting, each of the studies would have reported its data using the same metrics. This heterogeneity of data made a direct comparison of the papers difficult as each variable under the umbrella of HRV represents slightly different information. Additionally, some studies focused more on comparing stress between participant groups rather than in response to different scenarios [2].

One problem that arose when analyzing the data within each study was that in some papers, data was not reported in comparison to a baseline HRV reading. In others, the difference between baseline and event had to be calculated, and therefore, statistical significance was not able to be determined. Given this limitation, this review commented on the trend that the data followed and was able to show that overall, the HRV metrics examined responded similarly to the stress of a medical emergency.

In these studies, HRV was measured in stressful situations. Because the perception of stress is variable among individuals and because no two medical emergencies are exactly alike, HRV results are not able to be measured in a standardized setting. This is why this paper focused on general trends in HRV rather than only looking at statistically significant results to demonstrate the effects of responding to medical emergencies on HRV variables.

## Conclusions

After a review of the current published literature, it was found that HRV variables respond predictably in medical personnel to the stress of responding to a medical emergency. The RMSSD, SDNN, and LF/HF variables have the greatest amount of evidence, supporting their use as valid and reliable noninvasive metrics of stress. Measuring HRV has also been shown to be more sensitive and specific than measuring changes in heart rate, and the methods of obtaining HRV data can be more cost-effective and specific than previous methods to evaluate stress.
